# Effects of swimming on cognitive and health outcomes in older adults and insights into participation facilitators and barriers: a systematic review

**DOI:** 10.3389/fmed.2026.1839102

**Published:** 2026-07-13

**Authors:** Mirou Jaana, Danielle Cruise, Chloe Georges

**Affiliations:** 1 Telfer School of Management, University of Ottawa, Ottawa, ON, Canada; 2 Faculty of Medicine and Health Sciences, Université de Sherbrooke, Sherbrooke, QC, Canada

**Keywords:** cognitive impairment, health outcomes, older adults, physical activity, swimming, systematic review

## Abstract

**Purpose:**

Non-pharmacological interventions like physical activity may preserve cognition, slow its decline, and reduce behavioral/psychological symptoms associated with cognitive impairment (CI). This review synthesizes evidence on swimming’s effects on cognitive functioning in older adults and additionally reports on other health outcomes and the drivers, facilitators, and barriers for participation.

**Methods:**

We conducted a systematic review following the Preferred Reporting Items for Systematic Reviews and Meta-Analyses (PRISMA) guidelines. Four databases (Medline, APA PsycINFO, CINAHL, Scopus) were searched for studies on swimming, cognitive health and wellbeing, and dementia. English articles reporting impacts were included. A structured coding framework guided data extraction; two researchers coded the studies (81% agreement). Discrepancies were resolved by a tiebreaker.

**Results:**

Seventeen studies met the inclusion criteria (out of 3,915 articles), published between 2007 and 2019 across 11 countries. Two were randomized control trials (RCT), and the majority were mostly cross-sectional (6 studies) of moderate-high quality. Older adults with dementia were mostly considered in the studies, with the majority of interventions being instructor-led programs in public pools. Six studies reported improvement in cognitive functioning associated with swimming. Additional behavioral and psychological benefits (e.g., improved attention, depression, anxiety) were noted, with physiological improvements being the most consistently noted (e.g., cardiorespiratory fitness, strength). Reported drivers for participating in swimming included improving cognitive wellbeing, pleasure/fun, and clinician recommendations. Facilitators/barriers were mapped using the Socioecological Model. Participation was largely influenced by intrapersonal factors acting as both facilitators (e.g., swimming pleasure/enjoyment) and barriers (e.g., fluctuating psychological symptoms).

**Conclusion:**

Swimming is a non-pharmacological intervention that demonstrates promising benefits for older adults’ cognitive functioning in addition to other physiological and psychological benefits. It is especially valuable in countries/regions where climate conditions limit year-round outdoor activity. More rigorous and clearly defined research designs (e.g., defined intensity/duration/delivery approach) are needed to generate more robust evidence on differential benefits across CI severity levels.

## 1 Introduction

The number of older adults is expected to double globally, reaching 2.1 billion (16% of the population) by 2050 (1, 2). Cognitive impairment (CI) is an umbrella term used to refer to conditions characterized by deficits to cognitive functioning (e.g., memory, learning, ability to concentrate, etc.) (3). It is increasing rapidly among older adults (3, 4), with an estimated prevalence of 28.3% in North America (3). CI may range in severity from mild CI (e.g., noticeable challenges with cognition with daily functioning remaining stable) to more severe forms that interfere with daily life and activities (e.g., Alzheimer’s, Lewy Body dementia, vascular dementia, etc.) (5). Common risk factors for CI include age, genetics, smoking status, diet and physical activity level, among others (5, 6).

Generally, CI is progressive, and may gradually impact daily activities (5, 7). Over time, the care needs of a person with CI increase necessitating 24-h care for patients with severe impairments (e.g., in a long-term care home), which is costly and puts additional pressure on the healthcare system (4, 5, 8). Non-pharmacological interventions, such as physical activity can alleviate the behavioral, psychological, and physiological symptoms experienced by persons with CI (9, 10) and may be used as a first line of defense to address the needs of older adults living with these conditions. Specifically, physical activity is considered as a “treatment” for improving the symptoms associated with Alzheimer’s and related dementias (7), and maintaining a healthy lifestyle (e.g., exercising) has also shown to be a protective factor in cognitive aging (e.g., reducing deterioration) (11–13). Specific benefits of physical activity on cognition that have been discussed in the literature include, among others, reduced inflammatory cytokines and improved cerebrovascular health, and there have been calls to research the types, thresholds, and levels of physical activity necessary to promote health in older adults (14).

In general, it is recommended that older adults participate in 150–300 min of moderate-to-vigorous physical activity every week to reduce their risk of negative health outcomes and maintain functional independence (15, 16). Aerobic exercises, including swimming, are particularly important as they increase hippocampal and entorhinal volumes (i.e., improving processing speed, working memory, cognitive flexibility, etc.) and serum neurofilament concentrations (i.e., associated with better cognitive functioning), and reduce age-related grey and white matter loss that can lead to impaired cognitive functioning and motor control (17). Compared to land-based activity (where gravity causes more variability in blood flow), swimming significantly increases middle cerebral artery blood velocity, which improves cognition by allowing more blood with oxygen and nutrients to reach the brain (18). Water also exerts hydrostatic pressure on a person’s body which helps to push blood from the limbs back to the heart and brain, which helps to improve circulation and stabilize cerebral blood flow compared to land exercise (19). Furthermore, the inability to breathe freely during swimming (e.g., when under the water) creates brief periods of elevated carbon dioxide, which engages the brainstem and can influence attention and autonomic responses (20, 21). While some aerobic exercises can be challenging for older adults, swimming is considered a low-impact exercise modality (i.e., given the buoyancy which lowers the water’s impact) with a low risk of injury, which is often feasible for older adults (22), despite that its effects in terms of exhaustion may vary depending on the respective health status. Prior studies have discussed the potential benefits of swimming in addressing the behavioral, psychological, and physiological symptoms associated with CI (23, 24), its impacts on falls and older adults’ balance (25, 26), and its potential for older adults’ social engagement (13, 27). Yet, little is known on the breadth and strength of this evidence and the impacts of this non-pharmacological intervention on older adults.

When aiming to synthesize empirical evidence on a topic, a systematic review is considered the most appropriate methodology (28). While a number of systematic reviews have been published on physical activity in general among older adults (29–31), it remains unclear what specific exercises are effective and provide most benefits to this population (32). Two systematic reviews investigated the impacts of aquatic exercise on older adults’ *balance* (26) and the *risk factors for falls* (25). But to the best of our knowledge no prior study has investigated the evidence on swimming for older adults beyond these outcomes, particularly on cognitive functioning. This is especially relevant given the low physical and cognitive effort required for swimming (33), and its ease of administration. This paper addresses this area and presents the results of a systematic review that critically appraises and synthesizes interdisciplinary evidence on the impacts of swimming on older adults’ cognitive functioning, and reports on other health outcomes, as well as the drivers and facilitators/barriers to swimming observed in these studies. By examining these impacts and factors, this research can inform clinical practice, public health strategies, and policy efforts that promote healthy aging and evidence-based swimming interventions.

## 2 Methodology

Following the Preferred Reporting Items for Systematic Reviews and Meta-Analysis (PRISMA 2020 guideline) (34), we searched four databases (Medline, APA PsycINFO, CINAHL, Scopus) using a search strategy, which was developed in consultation with a librarian, and combined the domains of swimming, cognitive health, wellbeing, and dementia. The PRISMA checklist is presented in Supplementary File 1. Examples of the search terms included “swim*,” “cognitive declin*,” “cognitive deterioration*,” “dement*,” and “Alzheimer Disease,” which were combined using Boolean operators (i.e., and/or). Specific details of the search strategies used across all databases are included in Supplementary File 2. The databases were searched from inception to March 11, 2026. The inclusion criteria consisted of articles reporting results on the impacts of swimming (i.e., all types of swimming including program and non-program based) on cognition/cognitive wellbeing in older adults, with full-text available, and published in English (Table 1). Studies were included regardless of participant’s baseline cognitive status, as long as cognitive function was assessed as an outcome. This approach was used to capture both the therapeutic impacts of swimming in individuals with cognitive impairment as well as the potential preventive effects of swimming in maintaining cognitive function and reducing cognitive decline in healthy older adults. Other outcomes reported in the studies, in addition to the drivers and facilitators/barriers to swimming, were also extracted and presented in this review as additional evidence. Studies were stratified based on cognitive status (i.e., studies involving older adults with CI or without CI) to distinguish between therapeutic and preventive effects.

**TABLE 1:** Inclusion and exclusion criteria.

Parameter (46)	Inclusion criteria	Exclusion criteria
Population	▮ All older adults aged 50 + years of age	▮ Non-human populations (e.g., trials involving mice)
Intervention/Exposure	▮ Swimming (i.e., all types of swimming including program and non-program based)	▮ All other types of physical activity (i.e., articles that do not separately report on the impacts of swimming) ▮ Articles that refer to water immersion, but only involve standing in water
Comparator	▮ We did not exclude studies based on whether they had comparison/control groups.	
Outcome	▮ Cognition and cognitive health	▮ Studies that did not assess or report on participants’ cognition or cognitive health
Study Design	▮ Peer-reviewed studies published in English with the full text available	▮ Non-peer reviewed studies, studies published in languages other than English, no full text available

For the purpose of this review, swimming was defined as purposeful bodily movement (e.g., treading water, stroke-based limb movements such as freestyle, breaststroke, or backstroke), performed in water-based environments (e.g., pools, natural bodies of water, etc.) (35). Given that CI (e.g., Alzheimer’s Disease) can cause irregular or degraded motor patterns (36, 37), articles were included if they described an older adult who was attempting to propel themselves through water, or was using repeated/intentional limb movements producing directional/forward travel in water (regardless of whether coordinated stroke-based limb movements were used such as freestyle, breaststroke, or backstroke) (35); studies directly referring to the activity as swimming were included.

Articles were excluded from the review if they only reported on water immersion (e.g., standing in an upright position without treading water or completing water-based movements (38)). In line with other systematic reviews on older adults, we considered studies that reported findings on participants 50 years and older (39, 40), living with or without CI. Studies that did not present separate findings on older adults were excluded from this review (e.g., (41)). Those related to all other types of physical activity (i.e., articles that did not separately report on the impacts of swimming) or other mental illnesses (e.g., bipolar disorder, schizophrenia, etc.), or that reported on non-human populations (e.g., trials involving mice) were excluded [e.g., (42)].

**FIGURE 1:**
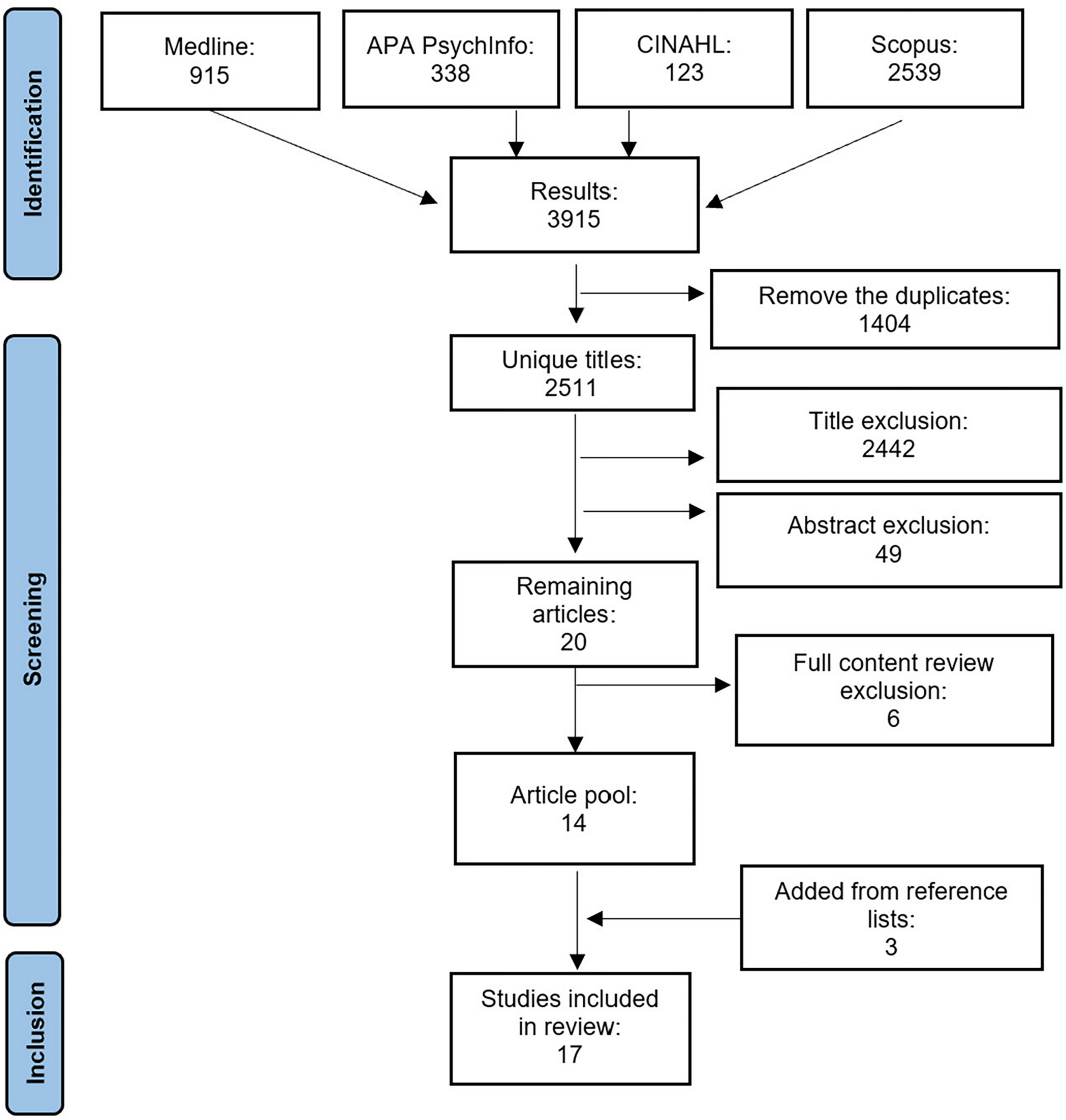
Flowchart presenting the review steps following the PRISMA guidelines.

The search resulted in 3,915 articles (Figure 1) that were reduced to 2,511 unique titles after removing duplicates (screening by DC and CG); Covidence was used to remove duplicates (43, 44). The abstracts of the remaining articles were further examined in line with the inclusion criteria, and a subset of 20 studies were kept for full text review. Six articles were further excluded for not meeting the inclusion criteria (e.g., two articles focused on swimmers who were young adults 18–25 years old (18, 45)). Three additional studies were added based on the review of the articles references lists leading to a total of 17 articles included in this review (Figure 1).

### 2.1 Data extraction

A coding scheme was developed to extract the relevant information from the studies, which included details on the study characteristics, type of CI and measures of cognition, swimming and reasons for swimming, location of the pool, time spent swimming, and the effects of swimming on older adults (i.e. quality of life, cognitive functioning, behavioral and psychological symptoms, physiological state, and functional ability). The facilitators and barriers to swimming were also identified and extracted from the empirical articles. Two researchers (DC and CG) completed the coding independently and the interrater agreement was 81%. Disagreements were resolved by a third researcher (MJ) as a tie breaker.

### 2.2 Level of evidence

We assessed the levels of evidence of the included studies using the Joanna Briggs Institute (JBI) guidelines (47). Within the JBI guidelines, studies are ranked based on the strength of evidence, with level 1 being associated with the strongest evidence (e.g., experimental designs) and level 5 with the weakest evidence (e.g., expert opinion and bench research) (47). These rankings are treated as categorical classifications, rather than continuous numerical scores, where the interpretation is based on the hierarchy of evidence defined by JBI, rather than the numerical value for each level of evidence. The levels of evidence are often used in medical research for readers to quickly understand how confident they can be in the findings of the study as higher levels of evidence (e.g., randomized control trials) may help reduce bias and/or confounders, while lower levels (e.g., non-experimental designs) of evidence may be informative but are generally less generalizable and/or prone to bias (48). Two researchers (DC and CG) extracted the information on the levels of evidence from the included articles.

### 2.3 Quality appraisal

In addition to the levels of evidence, the JBI has a suite of critical appraisal checklists that are designed to assess the methodological quality and study risk-of-bias, when relevant (49). These tools have been widely applied in the healthcare literature (50–52). To complete the quality appraisal, two researchers (DC and a research assistant) independently completed the JBI critical appraisal checklists for the relevant study type [i.e., cross-sectional studies (53), quasi-experimental studies (54), randomized control trials (55), qualitative research (56), and case reports (57)].

The critical appraisal tool for cross-sectional studies consists of 8 questions that focus on the methodological quality (i.e., clear inclusion criteria, measurement validity/reliability, identification/handling of confounders, and appropriate statistical analysis), as well as risk-of-bias that may arise from selection, measurement, or confounding (53). Similarly, the checklist for quasi-experimental studies assesses the methodological quality and risk-of-bias through 7 questions that cover 4 domains (i.e., cause-effect clarity, similarity of groups at baseline, risk-of-bias arising from selection, confounding, measurement, and completeness of follow-up) (54). The checklist for randomized control trials assesses both risk-of-bias and methodological quality across 5 domains (12 questions), including bias related to: selection and allocation, administration of intervention/exposure, assessment/detection/measurement of the outcome, participant retention, and statistical conclusion validity (55). The methodological quality of qualitative studies is assessed across 10 questions that consider the congruity of the research methodology and questions, research positioning and reflexivity, representation of the participants’ voices, ethical considerations, and methodological rigor (56). Last, the checklists for case reports consists of 8 questions used to assess the quality across 3 domains including description of the case (e.g., patient demographics/history), diagnostic methods/intervention details, and adverse events/takeaway lessons (57). Across all of the critical appraisal checklists, a standardized set of responses is used (i.e., Yes/Partial Yes/No/Unclear) (49). Interrater agreement was calculated (75%), and disagreements were resolved via consensus.

In line with other reviews, each item on the JBI quality appraisal checklists were scored as 1 (“yes”), 0.5 (“partial yes”), and 0 (“no” or “unclear”) to describe the overall quality of the included articles (58, 59). Total scores were converted into percentage. Studies meeting more than 75% of the criteria were classified as high quality; those meeting 51–75% as moderate quality; 26–50% as low quality; and 0–25% as critically low quality (58, 59). Given that excluding articles of critically low to moderate quality can introduce selection bias, omit evidence from “real-world” evaluations, and result in loss of information/data (60, 61), we opted to keep all articles that met the inclusion criteria, irrespective of their overall quality score. Our synthesis of the results and conclusions however takes into consideration the risk of bias and respective quality of the studies.

### 2.4 Data analysis and synthesis methods

Microsoft Excel was used to facilitate the data analysis process, with descriptive statistics (i.e., frequencies) and narrative summaries used to present the data. When relevant, tables and figures for data visualization were also created to present the data (62, 63). To describe the impacts of the swimming programs, we categorized the effects on older adults across five domains including quality of life (e.g., increased social interaction), cognitive functioning (e.g., improved memory and cognition), behavioral and psychological symptoms (e.g., reduction in behavioral problems), physiological condition (e.g., improved cardiovascular function), and functional ability (e.g., increased ability to complete activities of daily living). We reported the *p*-values and effect sizes, as applicable, in Table 2; in cases where the authors did not specify the significance of findings or effect sizes, we noted it in the table.

**TABLE 2:** Effects of swimming on older adults.

Participant’s cognitive impairment (CI) Status	Statistically significant	Not statistically significant	Not applicable
Quality of life (e.g., increased social interaction)			
Participants with CI (e.g., mild CI, dementia, Alzheimer’s, etc.)	• Group 1 (i.e., swimming and strength training) and group 2 (i.e., swimming and calisthenic exercise) had statistically significant improvements in their social resources (*p* = 0.023) (71)		• While statistical significance was not calculated, based on the Minimum Data Set 3.0 scores, the participant appeared to have increased ability to express himself, was more talkative and smiling more, and had less challenges finding words (83) • Only descriptive statistics were provided, although the authors suggest that neither the swimming or land-based activity groups appeared to have a greater effect on the participant’s orientation (i.e., environment, mood, and behaviours) (81)
Not specified (i.e., the article did not specify whether the participants had CI at baseline)		• There was no significant difference in social functioning in older masters swimmers versus younger masters swimmers • (*p* = 0.213, effect size = 0.09, measured using Cliff’s delta) (33)	
**Participant’s cognitive impairment (CI) Status**	**Statistically significant**	**Not statistically significant**	**Not applicable**
Cognitive Functioning (e.g., improved cognitive functioning)			
Participants with CI (e.g., mild CI, dementia, Alzheimer’s, etc.)	• Group 1 (i.e., swimming and strength training) and group 2 (i.e., swimming and calisthenic exercise) both had statistically significant improvements in cognitive capacity following the program, measured by the Mini-Mental State Exam scores (*p* = 0.034) (71) • Older adults who were swimmers performed significantly better than non-swimmers on two cognitive tests [i.e., category fluency (*p* = 0.005) and verbal N back test (*p* = 0.004)] (74) • The synchronized swimming group performed significantly better on the measures of delayed recall **(***p* = 0.005**)** (75) • Older adult swimmers performed significantly better on cognitive tests that assessed visuomotor coordination, motor persistence, attention, and response speed than non-swimmers (i.e., Digit Symbol Substitution test (*p* = 0.0401) and the Digit Vigilance test (*p* = 0.0036) (76)	• No differences were observed between the synchronized swimming and control groups on measures of executive function, naming, attention, language, abstraction, or orientation (*p*-values not reported) (75)	• The participant’s Mini Mental State Exam score improve from 2/30 to 4/30 following the aquatic intervention (83)
**Participant’s cognitive impairment (CI) Status**	**Statistically significant**	**Not statistically significant**	**Not applicable**
Participants without CI (e.g., healthy older adults)	• Significant changes were observed in the exercise group for attention (*p* = 0.002) (79) • Short-term exercising significantly increased executive functioning (*p* = 0.002), memory (*p* = 0.04), and overall performance (*p* = 0.001) (79) • Younger adult groups performed significantly better than the older adult groups on measures of cognitive function (i.e., speed of information processing: *p* = < 0.0001, Cohen’s *d* = 1.51; visual choice reaction time test: *p* = < 0.0001, Cohen’s *d* = 3.34; Stroop task: *p* = < 0.001, Cohen’s *d* = 1.10; Random Number Generation (RNG) task: *p* = < 0.02, Cohen’s *d* = 0.93; spatial running span task: *p* = < 0.0001, Cohen’s *d* = 2.23; dimension switching task: *p* = < 0.0003, Cohen’s *d* = 1.74; Stimulus Response Switching Task: *p* = 0.0031, Cohen’s *d* = 1.09). In the older adult group, regular swimming was significantly improved performance on measures of behavioral inhibition (i.e., RNG: *p* = < 0.011, Cohen’s *d* = 0.85), working memory updating (i.e., verbal running span: *p* = *p* < 0.0001, Cohen’s *d* = 1.45), and cognitive flexibility (i.e., Wisconsin card sorting test: *p* < 0.0001, Cohen’s *d* = 1.41; Stimulus response switching: *p* < 0.017, Cohen’s *d* = 0.76) (73)	• No significant differences were found between the three groups (i.e., sedentary, resistance training, and water-based exercise groups) on measures of sustained visual attention, learning ability, and working memory tests (*p*-values not reported) (77) • In the older adult group, regular swimming did not significantly improve information processing speed (*p* = 0.73) (73)	
Not specified (i.e., the article did not specify whether the participants had CI at baseline)		• There was no significant difference cognitive functioning factors between older masters swimmers and younger swimmers (i.e., memory: *p* = 0.407, effect size = 0.07; distractibility: *p* = 0.919, effect size = 0.01; blunders: *p* = 0.694, effect size = -0.04; names: *p* = .213, effect size = 0.09) (effect sizes measured using Cliff’s Delta) (33)	
**Participant’s cognitive impairment (CI) status**	**Statistically significant**	**Not statistically significant**	**Not applicable**
Behavioral and psychological symptoms (e.g., reduction in behavioral problems, etc.)			
Participants with CI (e.g., mild CI, dementia, Alzheimer’s, etc.)	• Group 1 (i.e., swimming and strength training) (*p* = 0.045) and group 2 (i.e., swimming and calisthenic exercise) (*p* = 0.041) had statistically significant improvements in their anxiety scores (71) • Statistically significant decline in psychological symptoms in persons with dementia were observed over the 12-week intervention (i.e., measured using the Psychological WellBeing in Cognitively Impaired Persons Scale (*p* = 0.034) and the Revised Memory and Behavior Problems Checklist (*p* = 0.001) (24)	• Revised Memory and Behavioral Problem Checklist (RMBPC) scores for the exercise vs. usual care groups approached significance (*p* = 0.06) between baseline and post-intervention (23)	
Participants without CI (e.g., healthy older adults)	• In the post-period, significantly improved psychological outcomes [i.e., depression (*p* < 0.05, partial eta effect size = 0.14), self-efficacy (*p* < 0.05, partial eta effect size = 0.15), decisional balance (*p* = < 0.005, partial eta effect size = 0.21)] were observed (72) • Compared to the control group, anxiety and depression levels were significantly improved following 6, 12, and 18 months of swimming (*p*s = < 0.01) (78)		
**Participant’s cognitive impairment (CI) Status**	**Statistically significant**	**Not statistically significant**	**Not applicable**
Not specified (i.e., the article did not specify whether the participants had CI at baseline)	• Measured using the Veterans RAND 12-Item Health Survey, older swimmers reported significantly better mental health than younger swimmers on two mental health items (i.e., item #1: *p* = < 0.001, effect size = 0.30; item #2: *p* = 0.007, effect size = 0.24) (effect sizes calculated using Cliff’s Delta) (33)	• Measured using the Veterans RAND 12-Item Health Survey there was no significant difference in the emotional factors of older and younger masters swimmers (i.e., *p* = 0.538, effect size = 0.05) (33)	
**Participant’s cognitive impairment (CI) status**	**Statistically significant**	**Not statistically significant**	**Not applicable**
Physiological effects (e.g., improved cardiovascular function)			
Participants with CI (e.g., mild CI, dementia, Alzheimer’s, etc.)	• Significant differences were observed between the exercise and control groups on measures of body mass index (*p* = 0.007, effect size = 0.162), skeletal mass index (*p* = 0.002, effect size = 0.220), lean mass (*p* = 0.001, effect size = 0.278), or grip strength (right hand *p* = 0.017, effect size = 0.137, left hand *p* = 0.003, effect size = = 0.218) post-intervention (23) • Group 1 (i.e., exercises in water and strength training) demonstrated statistically significant improvements in left (*p* = 0.042) and right *p* = 0.039) dynamometry, leg strength (*p* = 0.001), and abdominal strength (*p* = 0.046**)**, along with significantly reduced heart rate (*p* = 0.022) following the program. Both groups 1 and 2 (i.e., swimming and calisthenic exercise) demonstrated significantly improved balance (group 1: *p* = 0.035; group 2: *p* = 0.040) and flexibility (group 1: *p* = 0.016; group 2: *p* = 0.036) (71) • Post-intervention left-hand grip strength significantly improved (*p* = 0.017, effect size = (Wilcoxon analysis *r* = 0.53)) (80)	• No significant differences were observed between the exercise and control groups on measure of fat mass (*p* = 0.433, effect size = 0.015) (23) • No significant differences were found for percent body fat (*p* = 0.154), lean mass (*p* = 0.314), right-hand grip strength ( = 0.106), standing balance (*p* = 0.446), and the step test (Right: *p* = 0.748; Left: 0.864) post-swimming intervention (80)	
Participants without CI (e.g., healthy older adults)	• The water-based exercise group performed significantly better on the cardiorespiratory (*p* = < 0.01) and lower limb muscle strength tests (*p* = < 0.01) than the sedentary group (77) • Using the Rapid Assessment of Physical Activity and 2-min step test, a significant increase in cardiovascular fitness was observed in the exercise group post-intervention (*p* = 0.019), compared to the control group (*p* = 0.05) (79) • Significant improvements in VO_2_ max (i.e., cardiorespiratory fitness) (*p* = < 0.0001, partial eta effect size = 0.54) and heart rate variability (i.e., cardiac vagal control) (*p* < 0.05) were observed for the aquatic group post intervention compared to the sedentary older adults (72)		
**Participant’s cognitive impairment (CI) Status**	**Statistically significant**	**Not statistically significant**	**Not applicable**
Functional Ability (e.g., increased ability to complete activities of daily living)			
Participant’s cognitive impairment (CI) status	Statistically significant	Not statistically significant	Not applicable
Participants with CI (e.g., mild CI, dementia, Alzheimer’s, etc.)	• Group 2 (i.e., those participating in swimming and calisthenic exercise) had significantly improved functional level, specifically the ability to compete daily tasks (*p* = 0.040) (71)	• In the exercise vs. control groups, there were no statistically significant differences in the Seniors Physical Performance Battery (i.e., a measure of function comprised of standing balance, chair stands, and 2.4 m walk) (effect size = 0.149). Increased ability to complete activities of daily living was almost significant (effect size = 0.07) post-intervention (23)	
Participants without CI (e.g., healthy older adults)	• The water-based exercise group performed significantly better on the functional mobility test than the sedentary (*p* = < 0.01) and resistance training (*p* = < 0.05) groups. For reaction time (i.e., response and movement latencies), visual stimulus response time was significantly lower in the water-based group than the sedentary group (*p* = < 0.05) (77) • The latency, reaction times, and amplitude of the P2, N2, and P3 were significantly longer than those in the control group after the 12-month intervention period (*p* = < 0.01) (78)	• For reaction time (i.e., response and movement latencies), there were no significant difference in the visual stimulus response time for the water-based exercise and resistance training groups (*p*-values not reported) (77)	

To synthesize the facilitators/barriers, we used the Socioecological Model (SEM) (64, 65). The SEM originally developed for conceptualizing human development (64, 65) has been applied in the literature to understand the facilitators and barriers toward physical activity programs (66–68). It consists of five levels of factors that may act as facilitators or barriers to a person’s behavior, including: *intrapersonal factors* (i.e., those associated with the individual such as their attitudes, beliefs, skills, etc.); *interpersonal factors* (i.e., those related to a person’s close social circles and networks, such as friends, family, program instructors, etc.); *organizational factors* (i.e., those affecting an individual’s relationship with a single organization such as cultural and social norms, social institutions, etc.); *community factors* (i.e., those that affect a person’s relationship with their community and physical environment such as the design, connectedness, access to services/programs, etc.); and *public policy factors* (i.e., those related to the policies, laws, and regulations that are enforced at the national, provincial, and local levels) (66–68).

Given the high heterogeneity in the populations (e.g., older adults with/without CI, omission of details surrounding the participants CI status, etc.), intervention (e.g., differences in swimming programs in relation to frequency, intensity, duration, etc.), comparators (e.g., no control groups, treatment as usual as the control, different types of exercise as the control group, etc.), and reported outcomes (e.g., differences in tools/scales used to assess cognition), as well as the overall quality of the included articles, we opted for conducting a systematic review (rather than a meta-analysis), which is in line with the recommendations explaining the risk of inaccurate and invalid results if meta-analyses are conducted in these cases/conditions (69, 70). In line with this, a formal assessment of reporting bias (e.g., funnel plot or Egger’s test) or quantitative assessment of the certainty of evidence was also not performed given the heterogeneity previously described and the narrative nature of the synthesis. Potential reporting bias is noted qualitatively in terms of missing *p*-values and effect sizes in Table 2 and certainty of evidence was considered qualitatively in terms of the JBI Levels of Evidence and the quality appraisal throughout the reporting of findings and conclusions.

## 3 Results

### 3.1 General overview

**FIGURE 2:**
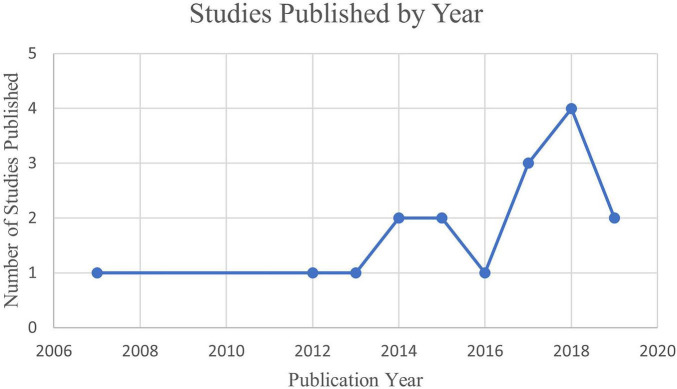
Time trend of published studies.

Table 3 presents an overview of the studies included in this review, which were published between 2007 and 2019 (Figure 2) in 9 countries including: United States (4 articles), Australia (4 articles), France (2 articles), India (2 articles), United Kingdom (1 article), Spain (1 article), China (1 article), Japan (1 article), and Brazil (1 article). Six studies used a cross-sectional or pre-post designs (35%, respectively), with quantitative methods/analyses. Most studies were published in medical journals (41%), followed by gerontology/aging journals (33%).

**TABLE 3:** Overview of the studies included in the review.

Authors (reference)	Country of origin	Main objective of the study	Study design and approach	Sample size		mean age	Type of cognitive impairment	Type of swimming	Location of the pool	Outcome of interest
				I	C					
Hobden et al. (82)	United Kingdom	To explore how people with dementia (and others involved) experienced a swimming and to define what ‘dementia friendly swimming’ is.	Interviews (Qualitative)	*N* = 23		Not indicated	Dementia	Program based led by staff	Public pool	The experiences of those with dementia in a swimming program
Bento-Torres et al. (77)	Brazil	To investigate the influence of resistance and water-based training on the cognitive function of healthy older adults	Cross sectional (Quantitative)	N (water based group) = 14	N (resistance training) = 14 N (sedentary) = 19	Sedentary group: 70.9 years, Resistance training: 71.7 years, Water-based 71.2 years	Healthy older adults, although Mini Mental State Exam scores may represent some CI among participants	Both individual and group-based swimming	N/A	The influence of two exercise modalities on cognitive function.
Becker and Lynch (84)	United States	To present a case study of a patient with end-stage Alzheimer’s disease and encourage further research that demonstrates the potential physiological improvements of swimming for those with end stage Alzheimer’s disease.	Case report (mixed methods)	N (swimming) = 1		53	Alzheimer’s Disease	Staff led program	Public pool	Functional and behavioral gains of a patient with end-stage Alzheimer’s disease after swimming
Geard et al. (33)	Australia	To test aging models to determine which models is appropriate for aging research on masters’ athletes. In addition, the authors aimed to compare the survey scores of younger and older masters swimmers.	Cross sectional (Quantitative)	*N* = 169	No control group	57.4 years	Not indicated, although cognition was assessed using the Cognitive Failures Questionnaire	Both individual and group-based swimming	N/A	Successful aging, psychological/social/ cognitive functioning, self-rated successful aging
Schilling et al. (81)	United States	To compare water and land activity on the behavior of persons with Alzheimer’s disease and other forms of cognitive impairment.	Single subject alternating treatment design (Quantitative)	*N* = 5	No control group	Ranged reported: 80–85 years	Dementia	Group program based called “friends swim club” led by instructor	N/A	Behavior mood or orientation
Vasegowda (76)	India	To study the effects of swimming on age related cognitive decline.	Cross sectional (Quantitative)	*N* = 50	*N* = 50	Participants were greater than 60 years old	“Natural” elderly cognitive decline	Individual	N/A	The relationship between swimming and cognition in older adults.
Henwood et al. (23)	Australia	To evaluate the physical and psychological benefits associated with participating in the Water memories swimming program.	Pre-post (Quantitative)	*N* = 23	*N* = 23	80.5 years for the intervention, 84.3 control group	Dementia	Program based led by trained staff with volunteer assistance	Public pool	Physical and psychological benefits of swimming for residents, feasibility and barriers to program delivery
Maeshima et al. (75)	Japan	To explore the cognitive functioning of middle aged and older adults who participate in synchronized swimming.	Cross sectional (Quantitative)	*N* = 23	*N* = 36	69.8 years for the intervention group, 67.0 years for the control	Participants were suspected to have mild cognitive impairment	Program based led by staff	Public pool	Cognition of adults who participate in synchronized swimming.
Varsha and Shashikala (74)	India	To study the effect of swimming on age related cognitive decline.	Cross sectional (Quantitative)	*N* = 40	*N* = 40	Participants were greater than 60 years old	Cognitive impairment in general	Individual	N/A	Cognitive functioning in older adults who swim compared to those who do not.
Albinet et al. (72)	France	To examine the effects of an aquatic aerobic exercise training program on measures of executive performance and their relationships with changes in cardiorespiratory fitness, cardiac vagal control and psychological variables	Randomized control trial (Quantitative)	N (swimming) = 19	N (stretching) = 17	Swimming group: 67 years Stretching group: 66 years	Healthy older adults (i.e., Mini Mental State Exam Scores greater than 26)	Staff led program	N/A	Cardiorespiratory fitness, cardiac vagal control (i.e., heart rate variability) and psychological variables (i.e., executive functioning) in sedentary older adults who participated in the study
Fedor et al. (79)	United States	To explore the effects of a water-based intervention on cardiovascular and cognitive functioning in healthy, community-dwelling older adults.	Pre-post assessment (Quantitative)	*N* = 27	*N* = 33	65.78 in the control group, 63.52 in the intervention group	Healthy older adults (i.e., Montreal Cognitive Assessment Scores greater than 23)	Program based led by certified trainers and lifeguards certified in first aid	N/A	Cardiovascular and cognitive results
Henwood et al. (80)	Australia	To explore what benefits (if any) there are for those with dementia living in nursing homes.	Pre-post assessment (Quantitative)	*N* = 24		88.4 years	Dementia	Program based guided by trained and qualified swimming instructor educated in the program, and assisted in the pool by program volunteers (facility staff or carer)	Public, municipal, indoor pool	Feasibility of the swimming program for residents of long-term care homes with dementia
Neville et al. (24)	Australia	To explore the effects of a swimming club for persons with dementia.	Pre-post assessment (Quantitative)	*N* = 11	No control group	88.4 years	Dementia	Instructor led program, volunteers to support	Public pool	Behavioral and psychological symptoms, psychological wellbeing of residents
Zhang et al. (78)	China	To observe the effects of different types of physical activity on cognitive functional decline and emotion in older adults on the P300.	Pre-post assessment (Quantitative)	*N* = 30	N (running) = 30 N (dancing) = 30 N (tai chi) = 30 N (control) = 30	Swimming group: 64.11, running group 65.00, dancing group 65.20, Tai chi group 65.5, and control group 64.10	Healthy older adults (i.e., excluded those with organic diseases neurology)	Individual	Public pool	Cognitive decline in older adults
Myers et al. (83)	United States	To investigate a case report of aquatic therapy for a patient that has advanced Alzheimer’s.	Case report (Quantitative)	*N* = 1	No control group	89 years	Alzheimer’s Disease	Program based led by staff	Public pool	The improvement in cognition, mood, and functional assessment after receiving aquatic therapy
Abou-Dest et al. (73)	France	To compare the performance of younger and older adults on various cognitive tasks (i.e., information processing speed, inhibition, working memory, updating, and shifting). The second objective was to determine the selectivity of the relationship between swimming and older adults.	Cross sectional (Quantitative)	N (active older adults) = 16	N (young adults) = 16 N (sedentary older adults) = 16	Young adult group: 23.56 Active older adult group: 69.13 Sedentary older adult group: 69.25	Healthy older adults (Mini Mental State Exam score greater than 25)	N/A	N/A	Impacts on executive (cognitive) functioning), Behavioral inhibition, working memory
Cancela Carral and Ayán Pérez (71)	Spain	To analyze the effects of a water-based program on older adults over a 5 week program.	Randomized control trial (Quantitative)	*N* = 31	*N* = 31	68.4 years	Possible mild cognitive impairment (Mini Mental State Examination score greater than or equal to 22)	Staff led program	Public pool	Impacts on condition, cognitive ability, function, and quality of life

N/A, Not indicated in the article. Sample Size: I, Intervention group; C, control/comparison group.

### 3.2 Level of evidence

Two studies were classified at level 1-experimental designs i.e. randomized control trials (highest level of evidence) (71, 72). The remaining mostly used a cross-sectional (6 articles) (level 4-observational descriptive studies) (33, 73–77) or quasi-experimental designs [i.e., 3 studies with a control group (23, 78, 79) and 3 without control groups (24, 80, 81) (level 2-quasi-experimental designs)]. One study used a qualitative research design (82), and two used a case report design (83, 84) (i.e., level 5-expert opinion and bench research).

There was a considerable variation in sample size in the studies included in this review [range: (1–169)]. Participants generally lived in their own home or in a long-term care home (41%, respectively); limited information was available on their living arrangement (e.g., alone, with friends/family, significant other, etc.). In seven articles (41%), the authors referred to dementia (23, 24, 80–82) or Alzheimer’s disease (83, 84) as the CI. Cognitive tests (e.g., Mini-Mental State Exam, Montreal Cognitive Assessment, etc.) were used for assessment/confirmation of CI diagnosis in 5 of these articles (23, 24, 80, 83); one article reported that the participant underwent a neurologic assessment that included a positron emission tomography scan to confirm their diagnosis (84). Two articles reported that the older adults had dementia but did not indicate how the participants were diagnosed (81, 82). Four articles (24%) included older adults that were suspected to have mild CI (e.g., based on participants’ scores on cognitive tests) (71, 74–76). Five studies only considered healthy older adults (i.e., excluding participants with CI) (72, 73, 77–79) and one article did not specify whether the participants had CI (33). Of the articles that reported on healthy older adults or did not specify whether the participants had CI, all used a cognitive test (e.g., Cognitive Failures Questionnaire) to evaluate cognitive functioning following participation in swimming (33, 72, 73, 77, 79).

Most articles (76%) involved swimming programs led by staff (e.g., certified lifeguards, trained swimming instructors, etc.) and were held in public pools (53%). Eight studies did not specify the location of the swimming exercises (47%).

### 3.3 Quality of included articles

Most articles included in this review were of moderate quality (53%) and four (23%) were considered of high quality (1 cross-sectional study, one quasi-experimental study, and 2 case reports) (Supplementary Material 3). The remaining were assessed as relatively low (three studies-18%) in quality based on the JBI quality appraisal checklists.

Of the *cross-sectional* (33, 73–77) *and quasi-experimental studies* (23, 24, 78–81), most articles used objective, standard criteria to assess the condition (e.g., as baseline, the participants’ cognitive impairment level was assessed using standardized criteria such as validated cutoffs on the Mini Mental State Exam) and *measured the outcomes* in a valid and reliable way (i.e., the impacts of the swimming programs were assessed using validated and reliable instruments). Nevertheless, this was not the case for the *measurement of the exposure/intervention*, which was missing from many of the studies (i.e., only partial details were presented in 4/6 cross-sectional studies, no details presented in 2 cross-sectional studies). Specifically, cross-sectional and quasi-experimental studies often lacked a clear definition and description of the respective swimming programs (e.g., including program duration, frequency, intensity, etc.) and did not consistently track participant attendance in swimming programs (e.g., using participant attendance logs). Furthermore, only one cross-sectional study clearly identified potential confounders and used strategies to manage them (i.e., controlling for relevant confounders such as education level). In the quasi-experimental studies, half did not use control groups and of those that did (50%) it was unclear whether the participants included in the comparison groups were receiving similar treatment/care to those in the intervention swimming group.

Details related to the randomization of participants and assessment/measurement of outcomes were missing from the two *randomized control trials* (71, 72). Neither article blinded participants to their treatment assignment (i.e., the participants knew whether they were in the swimming program), blinded the assessor to the treatment assignment (i.e., researcher/assessors were aware of the group allocations), or analyzed the participants’ results in the groups to which they were randomized. Both studies excluded participants who dropped out or did not complete the intervention and had no mention of an intention-to-treat analysis which would require conducting the analysis with participants in their groups regardless of program adherence.

The only *qualitative study* (82) included in this review had strong alignment between the research question/objectives and methods used, adequate representation of the participants’ voices (e.g., represented through the inclusion of participant quotes), and ethical conduct of the research (e.g., ethical approval from an appropriate research ethics body, informed consent to participate, etc.). Information related to the reflexivity of the researcher and an explicit statement of the researcher’s philosophical perspective (e.g., interpretivism) were missing from the article.

The two *case reports* (83, 84) were of high quality and reported on all 8 questions across the 3 domains including a description of the case (e.g., patient demographics/history), diagnostic methods/intervention details, and adverse events/takeaway lessons. For example, Becker and Lynch included specific details on the patient such as age, living situation, diagnosis, and medications taken, among other details (84). Furthermore, they included a description of why the participant joined the swimming program, as well as a clear description of the intervention with specific details on the swimming program, including the temperature of the pool, duration of the overall program and sessions, and swimming methods used (e.g., ascending a ramp of 50 feet, followed by treading water in an upright position), with no adverse events occurring (84).

### 3.4 Effects of swimming on older adults on cognitive functioning

The effects of swimming on older adults’ cognitive functioning and other health outcomes reported in the studies (i.e., behavioral and psychological symptoms, quality of life, physiological condition, and functional ability) are presented in Table 2 and described in further details below.

Six studies (35%) found a statistically significant relationship between swimming and *improvement in cognitive functioning*. Most used a cognitive test to measure cognition (e.g., Montreal Cognitive Assessment, Mini-Mental State Exam, etc.), and one RCT involving a staff-led swimming program for individuals with suspected mild CI reported a statistically significant increase in the cognitive scores on the Mini-Mental State Exam (*p* = 0.034), albeit a sample size (*n* = 31) (71). Myers et al. (83) also reported an increase in scores for a participant with Alzheimer’s Disease on this test from 2/30 at baseline to 4/30 at 3-months post evaluation.

Four studies assessed *participants’ attention* that is a component of participants’ cognitive functioning. There were differences noted in the swimming interventions (e.g., duration, frequency) and the reported results across studies, as well as variation in the tests used to assess attention. For example, a cross-sectional study with a sample size of 50 participants with general CI measured attention using the category fluency test and executive functioning using the Verbal N back test, and found statistically improved scores among swimming participants compared to the non-swimming group (i.e., *p*-value for attention = 0.005; *p*-value for cognition = 0.004) (74). Another cross-sectional study reported that the scores on the Digit vigilance test were significantly (*p* = 0.0036) better among an older adult group with general CI (*n* = 50) that participated in an individual swimming program over a year period (i.e., 513 s to complete the test in the swimming group versus 525 s for those in the non-swimming group) (76). Fedor et al. (79) also reported significant improvements in healthy older adults’ sustained attention composite score from baseline, compared to the control group, using the adaptive rate continuous performance test, although no duration for the staff-led swimming program was specified (*p* = 0.002). Last, only one cross-sectional study showed no differences in the attention scores among participants with mild CI in a staff-led swimming program compared to the control group using the Montreal Cognitive Assessment, but the study had a sample size and a short intervention period (*n* = 23, 6 days) (75).

### 3.5 Other reported outcomes observed

#### 3.5.1 Behavioral and psychological symptoms

Two RCT studies and two quasi-experimental studies further discussed the impacts of swimming on *psychological and behavioral symptoms* of older adults and reported statistically significant improvements (e.g., depression and self-efficacy) associated with swimming (24, 71, 72, 78). Of these, one RCT involving participants with suspected mild CI in a staff-led swimming program, and one pre-post study involving an individual-based swimming program for healthy older adults (i.e., no CI), found statistically significant improvements in *anxiety* on the Health Orientation Scale (*p* = 0.045) and Hamilton Anxiety Scale (*p* < 0.01), respectively (71, 78). Using the Psychological WellBeing in Cognitively Impaired Persons Scale and the Revised Memory and Behavior Problems, Neville et al. (24) also observed statistically significant decline (*p* = 0.001) in the frequency of psychological symptoms and behavioral problems in older adults with dementia who participated in an instructor-led swimming program (i.e., from a median of 2 behavioral problems in older adults to 0 at post-intervention) (24), which was corroborated by Henwood et al.’s (23) scores that approached significance on the same checklist (*p* = 0.06).

#### 3.5.2 Quality of life (QoL)

Evidence on the impacts of swimming on *QoL* was not strong, which may be due, in part, to the limited number of studies that examined these effects. Out of four studies that investigated the relationship between swimming and QoL, two reported descriptive statistics only (81, 83), and one cross-sectional study did not find significant impacts (33). Yet, one RCT involving older adults with suspected mild CI participating in a staff-led swimming program showed statistically significant improvements in social resources (e.g., increases in older adults’ social networks, friendships and social visits, etc.), measured using the Older Americans Resources and Services Multidimensional Functional Assessment Questionnaire Scale (e.g., scores improved from 3.15/6 to 4.16/6 following the swimming program, *p* = 0.023) (71).

#### 3.5.3 Physiological conditions

Six studies (35%) showed statistically significant improvements in the *physiological health* of participants following participation in a swimming program; three used pre-post designs, two were RCTs, and one was a cross-sectional study (23, 71, 72, 77, 79, 80). Significant improvements were noted in three studies that evaluated *cardiorespiratory fitness* using the VO_2_ max test and walk tests. For example, a RCT of older adults without CI who participated in a staff-led swimming program found an increase of 17.2% post-swimming intervention (*p* < 0.0001, partial eta effect size = 0.54) (72), whereas another cross-sectional study of healthy older adults who participated in both individual-based and group-based swimming programs noted that the swimming group walked 568.8 m in 6 min vs. 400.5 m for the sedentary group (*p* < 0.01) (77). An increase of mean from 94.74 steps to 106.37 steps was also noted post swimming interventions on a 2 min Step Test among healthy older adults that participated in staff-led program-based swimming (*p* = 0.01) (79); these findings were not consistent however for older adults living with dementia (80).

Statistically significant impacts were noted on measures of *body strength* with improvements in lower limb muscle strength reported in one RCT using the adaptable dynamometric platform for older adults with suspected mild CI (*p* = 0.001) (71) and one cross-sectional study using 30-s Chair Stand Test for older adults without CI (*p* < 0.01) (77). Improvements in the *left-hand grip* strength were also observed post-intervention using a Jamar dynamometer with an Effect size of (*r* = 0.53) (80).

#### 3.5.4 Functional ability

Five studies further examined the effects of swimming on the *functional ability* of older adults (29%), out of which three (RCT, pre-post, and cross-sectional studies) reported statistically significant effects (18%). The sole RCT that measured the ability of older adults’ with suspected mild CI to complete activities of daily living and functional ability found statistically significant improvements post-swimming (i.e., 7.31/8 vs. 7.88/8 scores post-intervention using the Instrumental Activities of Daily Living Scale, *p* = 0.040) (71). Amelioration in latency and reaction times were also observed in a cross-sectional study involving older adults without CI using the Reaction Time Test (i.e., 362.6 s for the swimming group vs. 438.4 s for the sedentary group, *p* < 0.05) (77).

### 3.6 Drivers for swimming engagement

The studies reported various drivers for the swimming intervention including the desire for improving cognitive wellbeing (41%), exercising (12%), pleasure/fun activity (12%), and recommendation from a medical practitioner (12%) and from family/friends (6%). Other reasons included lack of responsiveness to other pharmacological treatments (12%), involvement in other physical activity programs through recreation centers (12%), interest in finding friends with CI (6%), and participation in a research study (6%).

### 3.7 Facilitators and barriers to swimming

**FIGURE 3:**
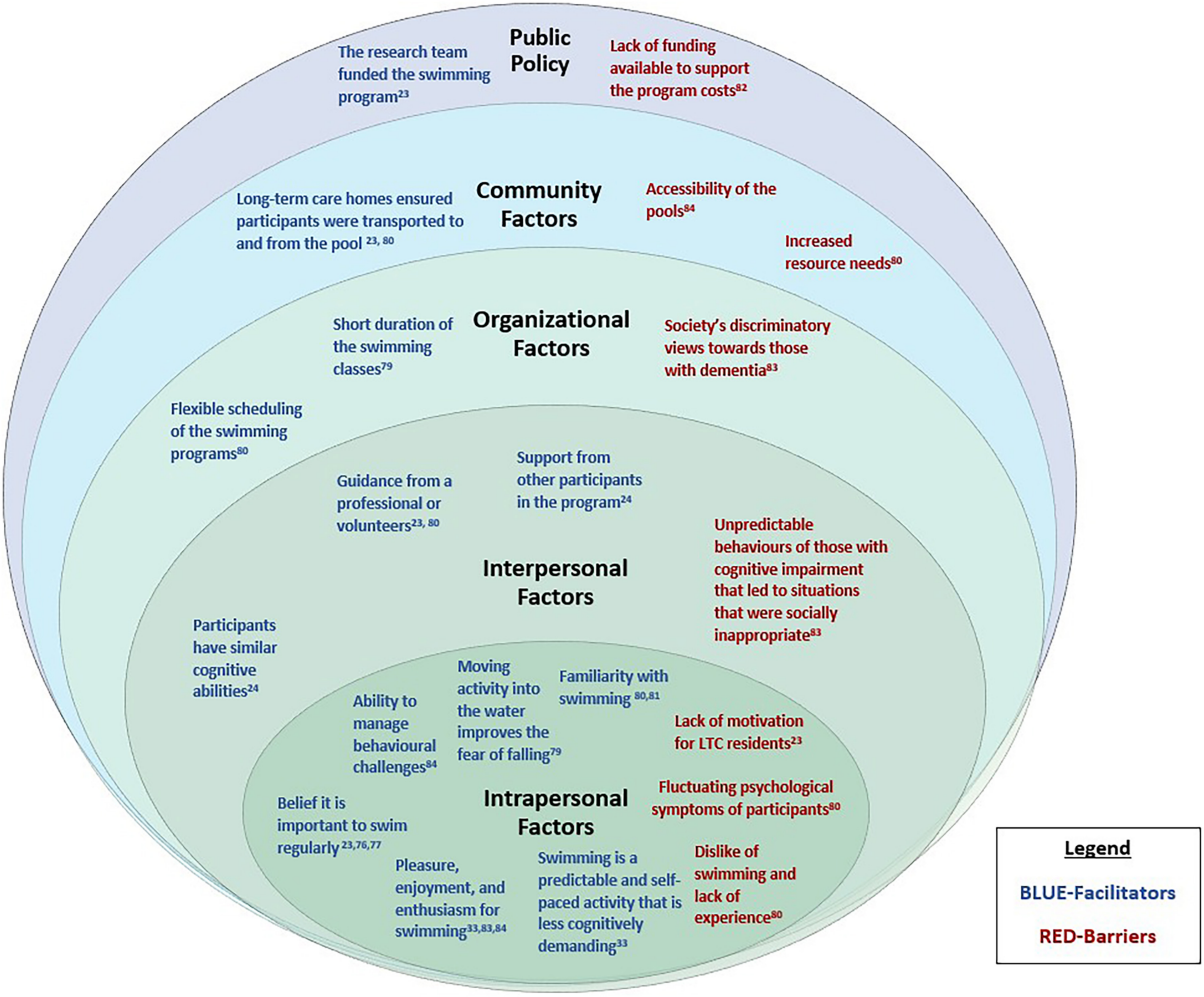
Facilitators and barriers to swimming categorized in the SEM.

Figure 3 summarizes the facilitators and barriers to swimming among older adults. A total of 10 studies (59%) presented information on facilitators/barriers, which we classified based on the Socioecological Model (SEM) (64, 65).

The *facilitators* identified in this review were mostly at the *intrapersonal level*, particularly in relation to the pleasure, enjoyment, and enthusiasm for participating in the swimming activity/intervention (33, 80, 82, 84). Others facilitators included the ability to manage the behavioral challenges associated with Alzheimer’s Disease (84), a reduction in participant’s fear of falling due to being in the water (79), familiarity with swimming (i.e., previous experience participating in swimming activities) (80, 81), and a belief that it is important to swim regularly to obtain the physical health benefits of swimming (23, 76, 77). Three studies that were conducted with participants from long-term care (LTC) homes, discussed facilitators at other levels including: the cost of the swimming program being funded by the research team as LTC homes may not have the funding to pay for residents to participate in swimming programs (*public policy level*) (23); LTC homes having the capacity to ensure that participants could be transported to and from the swimming pool (*community factors*) (23, 80); flexible scheduling of the swimming program (i.e., to allow residents to attend their meal times and other activities occurring within the LTC home) (*organizational factors*) (80); having staff led classes to provide guidance for safety and having participants with similar cognitive abilities participate in the swimming programs to facilitate social connections (*interpersonal level*) (23, 24, 80).

*Barriers* were discussed in only 24% of the studies involving swimming programs for older adults with CI (23, 80, 82, 84) and were mostly observed at the *intrapersonal level*: participants’ fear of water (84); fluctuating psychological symptoms of participants leading to behavioral challenges (e.g., disruptive behaviors) (80, 82); dislike of swimming or lack of previous swimming experience (80); and lack of motivation to participate in the swimming program (23). At the *interpersonal level*, the unpredictable behaviors of those living with dementia sometimes led to situations that were considered socially inappropriate (e.g., commenting on another person’s appearance in a negative manner) resulting in withdrawal of participants (82). The transport process from the care center to the pool (may cause increased agitation) and the limited accessibility to the pools (e.g., inability to climb up the pool ladder or stairs, need for a ramp to enter and exit the pool) can pose challenges to participation in swimming programs (*community factor*) (84). Interestingly, only one study identified barriers at the *public level*, which was associated with the cost and availability of funding for the swimming program (82).

## 4 Discussion

Physical activity presents health benefits in relation to cognitive function and physiological and psychological wellbeing. In recent years, we have seen a rise in research that highlights the impacts of aquatic exercising on cognitive functioning, particularly in individuals with neurodegenerative disorders (e.g., Dementia, Alzheimer’s disease, etc.) (24, 75, 76). Yet, little is known on the extent to which *regular swimming exercise can benefit older adults, particularly those living with CI*. This systematic review complements previous reviews on physical activity among older adults (25, 26) and synthesizes the research evidence on swimming for older adults in relation to cognitive function and other health outcomes.

An increasing trend in research publications was observed between 2016 and 2018; but surprisingly, since the pandemic, no recent studies were noted except for ones involving older mice and swimming activities in laboratory environments, which were not considered in this review (85–87). Although conducting laboratory work may inform conclusions on physiological impacts, it does not accurately depict real-life situations and actual living conditions that older adults face in the community and in LTC homes. A notable gap in the literature relates to the absence of studies conducted in countries/regions characterized by harsh winter climates (e.g., Canada, Scandinavian countries) where indoor swimming activities may represent an opportunity for older adults to remain physically active when outdoor exercise opportunities are limited.

**FIGURE 4:**
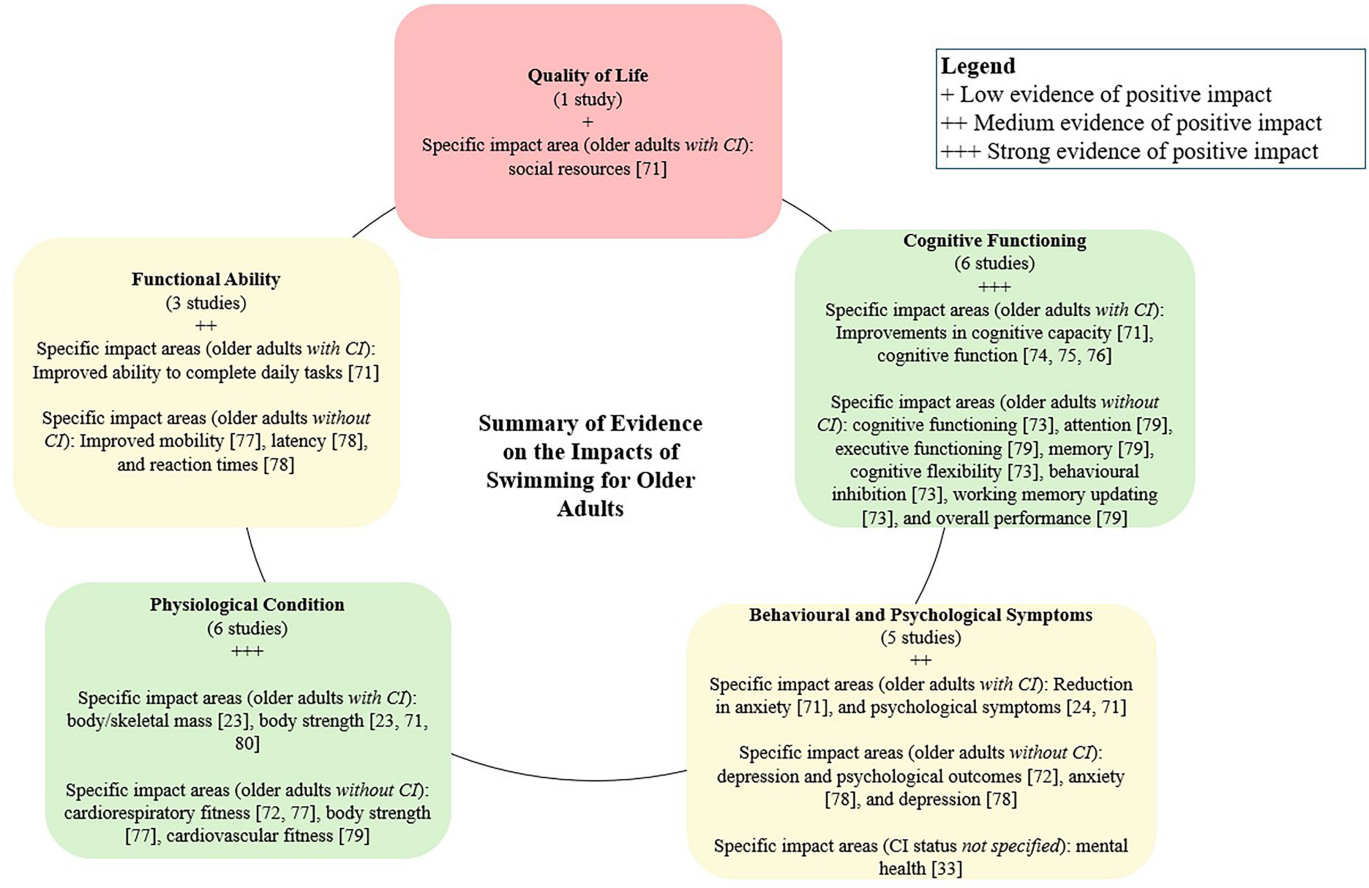
Summary of findings.

We categorized the level of evidence in Figure 4 based on the strength and consistency of the findings related to the impacts of swimming across the five outcomes domains (i.e., cognitive functioning, behavioral and psychological symptoms, quality of life, physiological condition, and functional ability). Specifically, *( + )* indicates low evidence of positive impact, reflecting limited or inconsistent findings, and/or a small number of studies. *(++)* denotes moderate evidence of positive impact, supported by a growing number of studies with generally consistent results. *(+++)* represents strong evidence of positive impact, characterized by a substantial body of literature and positive findings across multiple studies.

*Most studies included in this review identified statistically significant improvements in cognitive functioning, (e.g., improvements in cognitive test scores, etc.) and positive physiological impacts (e.g., increased VO_2_ max, cardiac vagal control, cerebral artery blood velocity, etc.) on older adults* (23, 71–77, 79, 80). Engaging in physical activity can lead to amelioration in blood circulation and oxygen transport to various parts of the body, including the brain, which plays a role in conserving the health of brain cells and decreasing the risk of cellular damage (88, 89). It also increases a person’s heart rate, blood flow, oxygen transportation, and nutrient delivery to the organs, and ensures that oxygen is delivered to the brain and cognition can effectively be supported (90). These findings are consistent with the literature on other groups in the population, which reported statistically significant improvements in VO_2_ max in swimmers when compared with a control group and positive effects on lung function in children and adolescents with chronic illnesses (91). Thus, we recommend the integration of swimming activities/programs, when feasible, as a regular non-pharmacological intervention and modality to support older adults cognitive and physiological functioning in long-term care and retirement homes, and potentially limit the fast development of frailty among this group.

In relation to the other health outcomes observed in the studies included in this review, they mostly focused on cardiovascular functioning, in specific; future research should investigate the impacts of swimming on other important physiologic measures (e.g., frailty, muscle atrophy etc.). Although a small number discussed the impacts of swimming on the quality of life, which may necessitate a longer follow-up duration to detect, a decline in anxiety and depression among older adults (with and without suspected CI) involved in swimming activities was noticed (71, 78). Neurotransmitters (i.e., dopamine, serotonin, and norepinephrine) and endorphins are usually released when a person exercises, which play a role in mood regulation, stress reduction, pain relief, and sense of contentment (92). Future studies should examine these outcomes and the conditions under which they are observed, to provide more conclusive evidence about the optimal programming of swimming activities.

To our surprise, limited information was presented on the living environment of participants included in the studies, which would be expected to relate to their activity level and overall wellbeing. In general, older adults do not participate in sufficient physical activity, which can lead to declining cognitive and physical health (93), and the setting in which they live may contribute to their limited activity (e.g., those living in residential care settings like long-term care and nursing homes) tend to exercise less (94). We recommend that future studies include a comprehensive description of the living environment of older adults and their health condition and consider this information in the analysis and reporting of findings.

The results of this review can inform LTC homes outreach and policy efforts to facilitate community initiatives for coordinated swimming activities that can benefit older adults (e.g., intergenerational programs, partnership with universities or community centers that have pools; public/private partnerships to develop indoor pools that are easily accessible for residents, etc.). LTC homes may have the capacity, as organizations, to develop and coordinate swimming programs that can improve the physical and psychological wellbeing of older adults, especially in countries with difficult winter climates that limit their ability to engage in outdoor exercises. Public/private partnerships and public health initiatives should focus on enabling access to indoor swimming pools and swimming programs available in local communities.

Most barriers and facilitators identified in this review appeared at the intrapersonal level (e.g., pleasure, enjoyment, enthusiasm for swimming) (33, 80, 82, 84). Although health decline associated with aging can act as a barrier to physical activity (66), empirical studies point to the fact that swimming is less cognitively and physically demanding compared to other activities, which facilitates older adults’ engagement in this exercise (24, 33). In addition, guidance from a professional (i.e., staff and volunteer led swimming programs) was noted as a facilitator to participating in swimming programs, which was particularly important when LTC residents were involved (e.g., as the residents had greater care needs and required additional assistance) (23, 24, 80). This may open the door to future intergenerational programs that leverage young, trained lifeguards and school volunteers who may provide community service and work as trainers for older adults (24, 33).

Only few studies investigated the barriers and facilitators to swimming programs at the public policy, community and organizational level factors. Given the findings in this review showing positive impacts of swimming (e.g., improved psychological and physiological states, reduced latency and reaction times, improved mobility), it is important to also explore these factors and initiate strategies that can facilitate a greater uptake of regular swimming among older adults in the community.

To evaluate the applicability of the evidence from the 17 empirical studies, the research team performed a *post-hoc* rating on a (7-point Likert Scale) to assess their usefulness to an older adult audience and their caregivers, along with their applicability to inform managers based on the subjective perceptions of the research team. The usefulness varied, with a median of 4/7 (range 2–6) and the median applicability to managers’ roles and decisions was 3/7 (range 2–6). These ratings were impacted by the limited information presented on the swimming programs/interventions presented in the studies, as it would be difficult for the reader/audience to understand and replicate a swimming program, for example, without a clear description of it. Thus, we call on future research and peer-reviewed journals to include a clear description of the swimming programs being proposed and provide sufficient details on them (e.g., type of swimming, location of the pool, time spent swimming, etc.) to understand the nature and magnitude of the “intervention” and improve the potential generalizability of the results. Adopting RCT designs with detailed intervention protocols (specifying frequency, duration, intensity, and type of swimming) published using guidelines like the Template for Intervention Description and Replication (TIDieR) (95) would contribute to raising the quality of evidence in this area.

### 4.1 Strengths and limitations

This systematic review adhered to the PRISMA 2020 guidelines (34) for comprehensive and unbiased reporting (although a protocol was developed, it was not registered in a public database). The literature search we conducted was developed based on existing literature and refined in consultation with a librarian, minimizing the risk of missing relevant studies. Nevertheless, it is important to note that we conducted our search for this SR on four databases using a search strategy that included articles only published in English, thus potentially excluding articles in other languages that may be relevant.

The quality assessment of the included studies was systematically performed using tools validated in the literature, which ensured a critical evidence appraisal. The quality of the studies was generally moderate constrained by missing information on statistical analyses used and the intervention/swimming program used, and the heterogeneity of the interventions and outcomes assessed (e.g., the duration of the program, frequency of the swimming sessions etc.). This limited our ability to compare across studies. Nevertheless, the positive pattern of impacts and significance across studies is an indication of potential benefits that swimming presents to older adults with and without CI. We recommend that peer-reviewed journals require that published studies include relevant essential details related to the intervention being studied and respective research environment to enable valid and rigorous evaluation of effects and a higher level of reported evidence.

## 5 Conclusion

Physical activity has been shown to have numerous benefits on older adults’ physical and psychological wellbeing (93). With the growing number of older adults (96), it is important to ensure that they have access to the care they need and prevent a fast deterioration in their cognitive abilities from an ethical perspective and health system performance standpoint.

Swimming programs present non-pharmacological interventions that can positively impact older adults cognitive functioning and other health outcomes, support community engagement, and reduce the burden on the health workforce by slowing their fast cognitive decline. This is particularly relevant in countries with harsh winter climates and available indoor public pools. We call on collaborative partnerships and policies that facilitate the use of community resources/pools and support intergenerational programs focused on swimming activities.

## Data Availability

The original contributions presented in the study are included in the article/Supplementary material, further inquiries can be directed to the corresponding author.

## References

[1] World Health Organization. *Ageing and Health.* (2021). Available online at: https://www.who.int/news-room/fact-sheets/detail/ageing-and-health (Accessed December 14, 2021).

[2] Amsalu ET Messele TA Adane M. Exploring the effect of professional experience on knowledge towards geriatric care among nurses working in adult care units. *BMC Geriatr*. (2021) 21:227. 10.1186/s12877-021-02156-3 33823796 PMC8025520

[3] Pais R Ruano L Carvalho OP Barros H. Global cognitive impairment prevalence and incidence in community dwelling older adults-a systematic review. *Geriatrics (Basel)*. (2020) 5:84. 10.3390/geriatrics5040084 33121002 PMC7709591

[4] Centers for Disease Control. *Cognitive Impairment: A Call for Action, Now!.* Atlanta: Centers for Disease Control (2011).

[5] The Alzheimer’s Association. 2023 Alzheimer’s disease facts and figures. *Alzheimers Dement*. (2023) 19:1598–695. 10.1002/alz.13016 36918389

[6] Xu Z Sun W Zhang D Chung VC Sit RW Wong SY. Comparative effectiveness of interventions for global cognition in patients with mild cognitive impairment: a systematic review and network meta-analysis of randomized controlled trials. *Front Aging Neurosci*. (2021) 13:653340. 10.3389/fnagi.2021.653340 34220484 PMC8249717

[7] Clina JG Bodde AE Chang J Helsel BC Sherman JR Vidoni. Factors associated with physical activity in Alzheimer’s disease: a cross-sectional study of individuals and their caregivers. *J Aging Health*. (2026) 38:148–58. 10.1177/08982643251318766 39894786 PMC12316988

[8] Comas-Herrera A Wittenberg R Pickard L Knapp M. Cognitive impairment in older people: future demand for long-term care services and the associated costs. *Int J Geriatr Psychiatry*. (2007) 22:1037–45. 10.1002/gps.1830 17603823

[9] Bherer L Erickson KI Liu-Ambrose T. Physical exercise and brain functions in older adults. *J Aging Res*. (2013) 2013:197326. 10.1155/2013/197326 24163767 PMC3791662

[10] Li YQ Yin ZH Zhang XY Chen ZH Xia MZ Ji LX et al. Non-pharmacological interventions for behavioral and psychological symptoms of dementia: a systematic review and network meta-analysis protocol. *Front Psychiatry*. (2022) 13:1039752. 10.3389/fpsyt.2022.1039752 36523873 PMC9744934

[11] Jung M Kim H Patrick Z Lee S. Health behaviors and executive function in late adulthood: a time-varying effect modeling analysis. *J Aging Health*. (2026) 38:159–68. 10.1177/08982643251319089 39901312

[12] Yu S Lin J Song S Huang S Liu F Xiao M. Understanding regular exercise behavior in frail older adults: a structural equation model based on social-cognitive variables. *BMC Geriatr*. (2025) 25:73. 10.1186/s12877-025-05702-5 39893383 PMC11786477

[13] Reitlo LS Sandbakk SB Viken H Aspvik NP Ingebrigtsen JE Tan X et al. Exercise patterns in older adults instructed to follow moderate- or high-intensity exercise protocol - the generation 100 study. *BMC Geriatr*. (2018) 18:208. 10.1186/s12877-018-0900-6 30200893 PMC6131829

[14] Izquierdo M Duque G Morley JE. Physical activity guidelines for older people: knowledge gaps and future directions. *Lancet Healthy Longev*. (2021) 2:e380–3. 10.1016/S2666-7568(21)00079-9 36098146

[15] Thornton JS Morley WN Sinha SK. Move more, age well: prescribing physical activity for older adults. *CMAJ*. (2025) 197:E59–67. 10.1503/cmaj.231336 39870409 PMC11771997

[16] Nilstomt A Gustavsson J Beckman L Bäccman C Nilson F Wagnsson S et al. Physical activity from the perspective of older adults: a convergent mixed-method study. *BMC Geriatr*. (2024) 24:768. 10.1186/s12877-024-05362-x 39294594 PMC11409466

[17] Serra L Petrosini L Mandolesi L Bonarota S Balsamo F Bozzali M et al. Walking, running, swimming: an analysis of the effects of land and water aerobic exercises on cognitive functions and neural substrates. *Int J Environ Res Public Health*. (2022) 19:16310. 10.3390/ijerph192316310 36498383 PMC9740550

[18] Shoemaker LN Wilson LC Lucas SJE Machado L Thomas KN Cotter JD. Swimming-related effects on cerebrovascular and cognitive function. *Physiol Rep*. (2019) 7:e14247. 10.14814/phy2.14247 31637867 PMC6803778

[19] Weenink RP Wingelaar TT. The circulatory effects of increased hydrostatic pressure due to immersion and submersion. *Front Physiol*. (2021) 12:699493. 10.3389/fphys.2021.699493 34349668 PMC8326965

[20] Paganini M Moon RE Camporesi EM Bosco G. Advances in breath-hold diving research: a state-of-the-art review. *Eur J Appl Physiol*. (2026) 126:1223–43. 10.1007/s00421-025-06093-6 41417060 PMC13013280

[21] Grossman KJ Lim DJ Murias JM Belfry GR. The effect of breathing patterns common to competitive swimming on gas exchange and muscle deoxygenation during heavy-intensity fartlek exercise. *Front Physiol*. (2021) 12:723951. 10.3389/fphys.2021.723951 34899369 PMC8652135

[22] Chen Y Lan Y Zhao A Wang Z Yang L. High-intensity interval swimming improves cardiovascular endurance, while aquatic resistance training enhances muscular strength in older adults. *Sci Rep*. (2024) 14:25241. 10.1038/s41598-024-75894-0 39448717 PMC11502738

[23] Henwood T Neville C Baguley C Beattie E. Aquatic exercise for residential aged care adults with dementia: benefits and barriers to participation. *Int Psychogeriatr*. (2017) 29:1439–49. 10.1017/S104161021700028X 28473006

[24] Neville C Henwood T Beattie E Fielding E. Exploring the effect of aquatic exercise on behaviour and psychological well-being in people with moderate to severe dementia: a pilot study of the Watermemories Swimming Club. *Australas J Ageing*. (2014) 33:124–7. 10.1111/ajag.12076 24521103

[25] Martínez-Carbonell Guillamón E Burgess L Immins T Martínez-Almagro Andreo A Wainwright TW. Does aquatic exercise improve commonly reported predisposing risk factors to falls within the elderly? A systematic review. *BMC Geriatr*. (2019) 19:52. 10.1186/s12877-019-1065-7 30795740 PMC6387499

[26] Kim Y Vakula MN Waller B Bressel E. A systematic review and meta-analysis comparing the effect of aquatic and land exercise on dynamic balance in older adults. *BMC Geriatr*. (2020) 20:302. 10.1186/s12877-020-01702-9 32842967 PMC7446104

[27] Li J Cai X Wamsiedel M. Perceptions, opportunities and barriers of social engagement among the Chinese older adults: a qualitative study. *BMC Geriatr*. (2024) 24:1033. 10.1186/s12877-024-05629-3 39716093 PMC11664919

[28] Paré G Trudel M-C Jaana M Kitsiou S. Synthesizing information systems knowledge: a typology of literature reviews. *Informat Manag.* (2015) 52:183–99. 10.1016/j.im.2014.08.008

[29] Koeneman MA Verheijden MW Chinapaw MJ Hopman-Rock M. Determinants of physical activity and exercise in healthy older adults: a systematic review. *Int J Behav Nutr Phys Act*. (2011) 8:142. 10.1186/1479-5868-8-142 22204444 PMC3320564

[30] Sun F Norman IJ While AE. Physical activity in older people: a systematic review. *BMC Public Health*. (2013) 13:449. 10.1186/1471-2458-13-449 23648225 PMC3651278

[31] Van Cauwenberg J De Bourdeaudhuij I De Meester F Van Dyck D Salmon J Clarys P et al. Relationship between the physical environment and physical activity in older adults: a systematic review. *Health Place*. (2011) 17:458–69. 10.1016/j.healthplace.2010.11.010 21257333

[32] Di Lorito C Long A Byrne A Harwood RH Gladman JRF Schneider S et al. Exercise interventions for older adults: a systematic review of meta-analyses. *J Sport Health Sci*. (2021) 10:29–47. 10.1016/j.jshs.2020.06.003 32525097 PMC7858023

[33] Geard D Rebar AL Reaburn P Dionigi RA. Testing a model of successful aging in a cohort of masters swimmers. *J Aging Phys Act*. (2018) 26:183–93. 10.1123/japa.2016-0357 28605264

[34] Page MJ McKenzie JE Bossuyt PM Boutron I Hoffmann TC Mulrow CD et al. The PRISMA 2020 statement: an updated guideline for reporting systematic reviews. *BMJ*. (2021) 372:n71. 10.1136/bmj.n71 33782057 PMC8005924

[35] Campbell J. *Swimming | Sports and Leisure | Research Starters | EBSCO Research.* (2024). Available online at: https://www.ebsco.com (Accessed April 22, 2026).

[36] Koppelmans V Silvester B Duff K. Neural mechanisms of motor dysfunction in mild cognitive impairment and Alzheimer’s disease: a systematic review. *J Alzheimers Dis Rep*. (2022) 6:307–44. 10.3233/ADR-210065 35891638 PMC9277676

[37] Poirier G Ohayon A Juranville A Mourey F Gaveau J. Deterioration, compensation and motor control processes in healthy aging, mild cognitive impairment and Alzheimer’s disease. *Geriatrics (Basel)*. (2021) 6:33. 10.3390/geriatrics6010033 33807008 PMC8006018

[38] Gentile A Vivirito S Kirkar M Paschos K Tuðan L Kulhánek J et al. Mindfulness training combined with cold water immersion effects on mood and perception of executive functioning in middle-aged and older adults: a pilot study. *Front Public Health*. (2025) 13:1693026. 10.3389/fpubh.2025.1693026 41283028 PMC12634357

[39] Huang Y Fleury J. Socially-supported sleep in older adults aged 50 and older: a concept analysis. *Front Public Health*. (2024) 12:1364639. 10.3389/fpubh.2024.1364639 38645458 PMC11027164

[40] Canneaux J Sharpe RA Orr N Smith JR Bethel A Phoenix C et al. Physical activity interventions for older adults - an overview of systematic reviews. *BMC Public Health*. (2026) 26:205. 10.1186/s12889-025-25002-2 41495724 PMC12805782

[41] Low L Graefe HLM Willcox-Pidgeon SM Barnett LM. Exploring the swimming and water safety behaviour among Indian and Vietnamese adults in Australia. *Health Promot J Austr*. (2026) 37:e70163. 10.1002/hpja.70163 41771673 PMC12953056

[42] Alomari MA Alzoubi KH Khabour OF. Swimming exercise improves short- and long-term memories: time-course changes. *Physiol Rep*. (2021) 9:e14851. 10.14814/phy2.14851 34110704 PMC8191402

[43] Macdonald M Martin Misener R Weeks L Helwig M. Covidence vs Excel for the title and abstract review stage of a systematic review. *JBI Evid Implement.* (2016) 14:200. 10.1097/01.XEB.0000511346.12446.f2

[44] McKeown S Mir ZM. Considerations for conducting systematic reviews: evaluating the performance of different methods for de-duplicating references. *Syst Rev*. (2021) 10:38. 10.1186/s13643-021-01583-y 33485394 PMC7827976

[45] Schaefer SY Louder TJ Foster S Bressel E. Effect of water immersion on dual-task performance: implications for aquatic therapy. *Physiother Res Int*. (2016) 21:147–54. 10.1002/pri.1628 25891889

[46] Methley AM Campbell S Chew-Graham C McNally R Cheraghi-Sohi S. PICO, PICOS and SPIDER: a comparison study of specificity and sensitivity in three search tools for qualitative systematic reviews. *BMC Health Serv Res*. (2014) 14:579. 10.1186/s12913-014-0579-0 25413154 PMC4310146

[47] Joanna Briggs Institute. *The JBI Approach: Levels of Evidence.* (2014). Available online at: https://jbi.global/sites/default/files/2019-05/JBI-Levels-of-evidence_2014_0.pdf (Accessed April 5, 2023)

[48] McNair P Lewis G. Levels of evidence in medicine. *Int J Sports Phys Ther* (2012) 7:474–81. 23091779 PMC3474306

[49] Joanna Briggs Institute. *Quality Appraisal Tools.* Adelaide, SA: Joanna Briggs Institute (2024).

[50] Farrah K Young K Tunis MC Zhao L. Risk of bias tools in systematic reviews of health interventions: an analysis of PROSPERO-registered protocols. *Syst Rev*. (2019) 8:280. 10.1186/s13643-019-1172-8 31730014 PMC6857304

[51] Quigley JM Thompson JC Halfpenny NJ Scott DA. Critical appraisal of nonrandomized studies-a review of recommended and commonly used tools. *J Eval Clin Pract*. (2019) 25:44–52. 10.1111/jep.12889 29484779

[52] Ma LL Wang YY Yang ZH Huang D Weng H Zeng XT. Methodological quality (risk of bias) assessment tools for primary and secondary medical studies: what are they and which is better? *Mil Med Res*. (2020) 7:7. 10.1186/s40779-020-00238-8 32111253 PMC7049186

[53] Barker TH Hasanoff S Aromataris E Stone JC Leonardi-Bee J Sears K et al. The revised JBI critical appraisal tool for the assessment of risk of bias for analytical cross-sectional studies. *JBI Evid Synth*. (2026) 24:401–8. 10.11124/JBIES-24-00523 40521701

[54] Barker TH Habibi N Aromataris E Stone JC Leonardi-Bee J Sears K et al. The revised JBI critical appraisal tool for the assessment of risk of bias for quasi-experimental studies. *JBI Evid Synth*. (2024) 22:378–88. 10.11124/JBIES-23-00268 38287725

[55] Barker TH Stone JC Sears K Klugar M Tufanaru C Leonardi-Bee J et al. The revised JBI critical appraisal tool for the assessment of risk of bias for randomized controlled trials. *JBI Evid Synth*. (2023) 21:494–506. 10.11124/JBIES-22-00430 36727247

[56] Lockwood C Munn Z Porritt K. Qualitative research synthesis: methodological guidance for systematic reviewers utilizing meta-aggregation. *Int J Evid Based Healthc*. (2015) 13:179–87. 10.1097/XEB.0000000000000062 26262565

[57] Moola S Munn Z Tufanaru C Aromataris E Sears K Sfetcu R et al. Chapter 7: Systematic reviews of etiology and risk. In: Aromataris E Munn Z editors. *Joanna Briggs Institute Reviewer’s Manual*. Adelaide, SA: The Joanna Briggs Institute (2017).

[58] Siddiqui AA Alshammary F Mulla M Al-Zubaidi SM Afroze E Amin J et al. Prevalence of dental caries in Pakistan: a systematic review and meta-analysis. *BMC Oral Health*. (2021) 21:450. 10.1186/s12903-021-01802-x 34530810 PMC8447584

[59] Woldegeorgis BZ Anjajo EA Korga TI Yigezu BL Bogino EA Tema HT et al. Ethiopians’ knowledge of and attitudes toward epilepsy: a systematic review and meta-analysis. *Front Neurol*. (2023) 14:1086622. 10.3389/fneur.2023.1086622 36925943 PMC10011168

[60] Harvey LA Dijkers MP. Should trials that are highly vulnerable to bias be excluded from systematic reviews? *Spinal Cord*. (2019) 57:715–6. 10.1038/s41393-019-0340-y 31492940

[61] Jia RM Stern C. The inclusion or exclusion of studies based on critical appraisal results in JBI qualitative systematic reviews: an analysis of practices. *Res Synth Methods.* (2026) 17:277–92. 10.1017/rsm.2025.10042 41635944 PMC12873616

[62] South E Rodgers M. Data visualisation in scoping reviews and evidence maps on health topics: a cross-sectional analysis. *Syst Rev*. (2023) 12:142. 10.1186/s13643-023-02309-y 37587522 PMC10433592

[63] Pollock D Peters MDJ Khalil H McInerney P Alexander L Tricco AC et al. Recommendations for the extraction, analysis, and presentation of results in scoping reviews. *JBI Evid Synth*. (2023) 21:520–32. 10.11124/JBIES-22-00123 36081365

[64] Bronfenbrenner U. Ecological systems theory. In: Vasta R editor. *Six Theories of Child Development: Revised Formulations and Current Issues.* London, England: Jessica Kingsley Publishers (1992). p. 187–249, 285.

[65] Bronfenbrenner U. Ecology of the family as a context for human development: Research perspectives. *Dev Psychol.* (1986) 22:723–42. 10.1037/0012-1649.22.6.723

[66] Bethancourt HJ Rosenberg DE Beatty T Arterburn DE. Barriers to and facilitators of physical activity program use among older adults. *Clin Med Res*. (2014) 12:10–20. 10.3121/cmr.2013.1171 24415748 PMC4453303

[67] Eyre ELJ Adeyemi LJ Cook K Noon M Tallis J Duncan M. Barriers and facilitators to physical activity and FMS in children living in deprived areas in the UK: qualitative study. *Int J Environ Res Public Health*. (2022) 19:1717. 10.3390/ijerph19031717 35162741 PMC8835542

[68] Martínez-Andrés M Bartolomé-Gutiérrez R Rodríguez-Martín B Pardo-Guijarro MJ Garrido-Miguel M Martínez-Vizcaíno V. Barriers and facilitators to leisure physical activity in children: a qualitative approach using the socio-ecological model. *Int J Environ Res Public Health*. (2020) 17:3033. 10.3390/ijerph17093033 32349290 PMC7246675

[69] Soni KD. When do we need to do meta-analysis!!! *Indian J Anaesth*. (2023) 67:673–4. 10.4103/ija.ija_333_23 37693030 PMC10488575

[70] Lensen S. When to pool data in a meta-analysis (and when not to)? *Fertil Steril*. (2023) 119:902–3. 10.1016/j.fertnstert.2023.03.015 36948444

[71] Cancela Carral JM Ayán Pérez C. Effects of high-intensity combined training on women over 65. *Gerontology*. (2007) 53:340–6. 10.1159/000104098 17575465

[72] Albinet CT Abou-Dest A André N Audiffren M. Executive functions improvement following a 5-month aquaerobics program in older adults: role of cardiac vagal control in inhibition performance. *Biol Psychol*. (2016) 115:69–77. 10.1016/j.biopsycho.2016.01.010 26812613

[73] Abou-Dest A Albinet CT Boucard G Audiffren M. Swimming as a positive moderator of cognitive aging: a cross-sectional study with a multitask approach. *J Aging Res*. (2012) 2012:273185. 10.1155/2012/273185 23326664 PMC3541603

[74] Varsha S Shashikala K. Effect of swimming on cognition in elderly. *Int J Physiol.* (2017) 5:94. 10.5958/2320-608X.2017.00063.4

[75] Maeshima E Okumura Y Tatsumi J Tomokane S Ikeshima A. Cognitive function in middle-aged and older adults participating in synchronized swimming-exercise. *J Phys Ther Sci*. (2017) 29:148–51. 10.1589/jpts.29.148 28210062 PMC5300828

[76] Vasegowda S. Swimming helps elderly population to improve mental speed and attention. *Int J Clin Exp Physiol.* (2018) 5:200–2. 10.5530/ijcep.2018.5.4.22

[77] Bento-Torres NVO Bento-Torres J Mendonça Tomás A Torres de Souza LG Oliveira de Freitas J Anderson dos Santos Pantoja J et al. Water based exercise and resistance training improve cognition in older adults. *Revista Brasileira de Medicina do Esporte.* (2019) 25:71–5. 10.1590/1517-869220192501190627

[78] Zhang X Ni X Chen P. Study about the effects of different fitness sports on cognitive function and emotion of the aged. *Cell Biochem Biophys*. (2014) 70:1591–6. 10.1007/s12013-014-0100-8 24997050

[79] Fedor A Garcia S Gunstad J. The effects of a brief, water-based exercise intervention on cognitive function in older adults. *Arch Clin Neuropsychol*. (2015) 30:139–47. 10.1093/arclin/acv001 25638041

[80] Henwood T Neville C Baguley C Clifton K Beattie E. Physical and functional implications of aquatic exercise for nursing home residents with dementia. *Geriatr Nurs*. (2015) 36:35–9. 10.1016/j.gerinurse.2014.10.009 25453191

[81] Schilling ML Coles R Simons C Frost R. Perceived benefits of an aquatic activity program on the behaviors of those with memory impairments: a pilot study. *Activ Adapt Aging.* (2018) 42:292–304. 10.1080/01924788.2017.1406837

[82] Hobden T Swallow M Beer C Dening T. Swimming for dementia: an exploratory qualitative study: innovative practice. *Dementia (London)*. (2019) 18:776–84. 10.1177/1471301218768372 29600876

[83] Myers KW Capek D Shill H Sabbagh M. Aquatic therapy and Alzheimer’s disease. *Ann Long Term Care.* (2013) 21:36–41.

[84] Becker BE Lynch S. Case report: aquatic therapy and end-stage dementia. *PM R*. (2018) 10:437–41. 10.1016/j.pmrj.2017.09.001 28918118

[85] Badriyah Hidayati H Sri Rejeki P Herawati L Wahyuning Asih S. *Swimming Improves Memory Function and Decreases N-Methyl-D-Aspartate in Ageing Rats.* ResearchGate (2024). 10.37506/ijfmt.v15i4.16883

[86] Bashiri H Enayati M Bashiri A Salari AA. Swimming exercise improves cognitive and behavioral disorders in male NMRI mice with sporadic Alzheimer-like disease. *Physiol Behav*. (2020) 223:113003. 10.1016/j.physbeh.2020.113003 32522682

[87] De Sousa RAL Diniz-Magalhaes CO Cruz PP de Oliveira GHB Prates JTAC de Azevedo Ferreira CM et al. Physical exercise inhibits cognitive impairment and memory loss in aged mice, and enhances pre- and post-synaptic proteins in the hippocampus of young and aged mice. *Neuromolecular Med*. (2024) 26:31. 10.1007/s12017-024-08798-x 39073519

[88] Cotman CW Berchtold NC Christie LA. Exercise builds brain health: key roles of growth factor cascades and inflammation. *Trends Neurosci*. (2007) 30:464–72. 10.1016/j.tins.2007.06.011 17765329

[89] Erickson KI Kramer AF. Aerobic exercise effects on cognitive and neural plasticity in older adults. *Br J Sports Med*. (2009) 43:22–4. 10.1136/bjsm.2008.052498 18927158 PMC2853472

[90] Nystoriak MA Bhatnagar A. Cardiovascular effects and benefits of exercise. *Front Cardiovasc Med*. (2018) 5:135. 10.3389/fcvm.2018.00135 30324108 PMC6172294

[91] Lahart IM Metsios GS. Chronic physiological effects of swim training interventions in non-elite swimmers: a systematic review and meta-analysis. *Sports Med*. (2018) 48:337–59. 10.1007/s40279-017-0805-0 29086218

[92] Basso JC Suzuki WA. The effects of acute exercise on mood, cognition, neurophysiology, and neurochemical pathways: a review. *Brain Plast*. (2017) 2:127–52. 10.3233/BPL-160040 29765853 PMC5928534

[93] Langhammer B Bergland A Rydwik E. The importance of physical activity exercise among older people. *Biomed Res Int*. (2018) 2018:7856823. 10.1155/2018/7856823 30627571 PMC6304477

[94] Douma JG Volkers KM Engels G Sonneveld MH Goossens RHM Scherder EJA. Setting-related influences on physical inactivity of older adults in residential care settings: a review. *BMC Geriatr*. (2017) 17:97. 10.1186/s12877-017-0487-3 28454563 PMC5408383

[95] Hoffmann TC Glasziou PP Boutron I Milne R Perera R Moher D et al. Better reporting of interventions: template for intervention description and replication (TIDieR) checklist and guide. *BMJ*. (2014) 348:g1687. 10.1136/bmj.g1687 24609605

[96] Eshkoor SA Hamid TA Mun CY Ng CK. Mild cognitive impairment and its management in older people. *Clin Interv Aging*. (2015) 10:687–93. 10.2147/CIA.S73922 25914527 PMC4401355

