# Supplementary material for: Effects of swimming on cognitive and health outcomes in older adults and insights into participation facilitators and barriers: a systematic review
Source: Front Med (Lausanne). 2026 Jul 13;13:1839102. doi: 10.3389/fmed.2026.1839102 (PMC13402527; doi:10.3389/fmed.2026.1839102)
Supplement: Supplementary file 2 [file Data_Sheet_2.pdf]

Additional File 1. Details of the Search Terms.

OVID Medline Search Terms

1 exp Dementia/ or exp "Mental Status and Dementia Tests"/ or exp Frontotemporal Dementia/ or exp Dementia, Vascular/ or exp Dementia, Multi-Infarct/

2 exp Cognitive Dysfunction/

3 exp Alzheimer Disease/

4 Dement*.ti,ab.

5 exp Cognitive Impairment/ or exp Alzheimer's Disease/

6 cognitive impairment*.ti,ab.

7 cognitive declin*.ti,ab.

8 mild neurocognitive disorder*.ti,ab.

9 mental deterioration*.ti,ab.

10 cognitive dysfunction*.ti,ab.

11 1 or 2 or 3 or 4 or 5 or 6 or 7 or 8 or 9 or 10

12 exp Swimming/

13 swim*.ti,ab.

14 aqua* fit*.ti,ab.

15 (water adj2 (exercis* or activit* or aerobic*)).ti,ab.

16 aquatic therap*.ti,ab.

17 12 or 13 or 14 or 15 or 16

18 11 and 17

APA PsychINFO Search Terms

1 exp Dementia/ or exp "Mental Status and Dementia Tests"/ or exp Frontotemporal Dementia/ or exp Dementia, Vascular/ or exp Dementia, Multi-Infarct/

2 exp Cognitive Dysfunction/

3 exp Alzheimer Disease/

4 Dement*.ti,ab.

5 exp Cognitive Impairment/ or exp Alzheimer's Disease/

6 cognitive impairment*.ti,ab.

7 cognitive declin*.ti,ab.

8 mild neurocognitive disorder*.ti,ab.

9 mental deterioration*.ti,ab.

10 cognitive dysfunction*.ti,ab.

11 1 or 2 or 3 or 4 or 5 or 6 or 7 or 8 or 9 or 10

12 exp Swimming/

13 swim*.ti,ab.

14 aqua* fit*.ti,ab.

15 (water adj2 (exercis* or activit* or aerobic*)).ti,ab.

16 aquatic therap*.ti,ab.

17 12 or 13 or 14 or 15 or 16

18 11 and 17

CINAHL Search Terms

((MH "Swimming") OR (swim*) OR (aquafit*) OR (aquatic therap*) OR (water N2 exercise*) OR (aquatic*)) AND (((MH dementia+) OR (MH Alzheimer’s disease+)) OR (dement* OR “cognitive impairment*” OR “alzheimer*” OR "cognitive declin*" OR "mild neurocognitive disorder*" OR "cognitive dysfunction*" OR “mental deterioration*" “mild dementia” OR “moderate dementia”))

SCOPUS Search Terms

(TITLE-ABS-KEY(dement* OR alzheimer* OR "cognitive impairment*" OR "cognitive declin*" OR "cognitive dysfunction*" OR "mild neurocognitive disorder*" OR "mental deterioration*" OR "Alzheimer disease" OR "Alzheimer's disease")) AND (TITLE-ABS-KEY(swim* OR aquafit* OR "aquatic therap*" OR aquatic*)
OR (water W/2 exercis*) OR (water W/2 activit*) OR (water W/2 aerobic*)
OR ("water-based exercise*"))
